# Reactive Oxygen Species are Essential for Placental Angiogenesis During Early Gestation

**DOI:** 10.1155/2022/4290922

**Published:** 2022-06-01

**Authors:** Yike Yang, Huili Jin, Yuhan Qiu, Yamin Liu, Li Wen, Yong Fu, Hongbo Qi, Philip N. Baker, Chao Tong

**Affiliations:** ^1^State Key Laboratory of Maternal and Fetal Medicine of Chongqing Municipality, The First Affiliated Hospital of Chongqing Medical University, Chongqing 400016, China; ^2^Department of Obstetrics, Peking University Third Hospital, Beijing 100191, China; ^3^Department of Obstetrics, The Affiliated Hospital of Guizhou Medical University, Guiyang 550004, China; ^4^Department of Obstetrics and Gynecology, The Second Affiliated Hospital of Chongqing Medical University, Chongqing 400010, China; ^5^Department of Obstetrics, Women and Children's Hospital of Chongqing Medical University, Chongqing 401147, China; ^6^College of Life Sciences, University of Leicester, Leicester LE1 7RH, UK

## Abstract

**Background:**

Preeclampsia (PE) is associated with insufficient placental perfusion attributed to maldevelopment of the placental vasculature. Reactive oxygen species (ROS) are implicated in angiogenesis, but their regulatory effects and mechanisms in placental vascular development remain unclear.

**Methods:**

Placental oxidative stress was determined throughout gestation by measuring 4-hydroxynonenal (4HNE) and malondialdehyde (MDA). The antioxidant MitoQ was administered to pregnant mice from GDs 7.5 to 11.5; placental morphology and angiogenesis pathways were examined on GDs 11.5 and 18.5. Moreover, we established a mouse mFlt-1-induced PE model and assessed blood pressure, urine protein levels, and placental vascular development on GDs 11.5 and 18.5. Human umbilical vein endothelial cells (HUVECs) were treated with various H_2_O_2_ concentrations to evaluate cell viability, intracellular ROS levels, and tube formation capability. MitoQ, an AKT inhibitor and an ERK1/2 inhibitor were applied to validate the ROS-mediated mechanism regulating placental angiogenesis.

**Results:**

First-trimester placentas presented significantly higher MDA and 4HNE levels. MitoQ significantly reduced the blood vessel density and angiogenesis pathway activity in the placenta on GDs 11.5 and 18.5. Serum sFlt-1 levels were elevated, and we observed poor placental angiogenesis and PE-like symptoms in cases with mFlt-1 overexpression. Moderate H_2_O_2_ treatment promoted HUVEC proliferation and angiogenesis, whereas these improvements were abolished by MitoQ, AKT inhibitor, or ERK1/2 inhibitor treatment.

**Conclusions:**

Moderate ROS levels are essential for placental angiogenesis; diminishing ROS with potent antioxidants during placentation decreases placental angiogenesis and increases PE risk. Therefore, antioxidant therapy should be considered carefully for normal pregnant women during early gestation.

## 1. Introduction

The placenta is an ephemeral organ that only exists during gestation and forms the feto-maternal interface. In addition to creating a stable milieu isolated from maternal and environmental stressors, the placenta is responsible for transporting substances between the mother and fetus to meet the requirements of fetal development. The dense networks of blood vessels within the placenta are responsible for exchanging gases, nutrients, and waste between mother and fetus throughout pregnancy, which is essential for proper fetal growth [1]. Abnormal development of the placental vasculature may lead to placental insufficiency, which is characterized by a poor uterine condition and results in various pregnancy complications for both the mother and fetus, including preeclampsia (PE) [2], fetal growth restriction (FGR) [3], stillbirth [4], or miscarriage [5].

Two blood vascular systems have been identified in the placenta. In early gestation, chorionic villi are essential structural and functional components of the human placenta. The mesenchymal core of villi is covered by a two-layered trophoblast epithelium. The inner layer is constituted by cytotrophoblast cells, which proliferate and differentiate through fusion, forming a multinucleated syncytiotrophoblast (STB) that covers the entire surface of the villus and is in direct contact with maternal blood. During the first and second trimesters, highly invasive extravillous cytotrophoblasts invade the uterine interstitium, while maternal spiral arteries are remodeled into uteroplacental arteries. Extravillous cytotrophoblasts, which adopt an endothelial phenotype, replace the maternal arterial endothelium [6–8]. On the other hand, fetal capillary segments are formed by vasculogenesis in early placental villi and then fuse and elongate to comprise a capillary network that expands by branching angiogenesis [8–10]. Angiogenesis begins 21 days after conception and continues throughout human gestation [11].

Fluctuations in the oxygen supply contribute to increased reactive oxygen species (ROS) production in the placenta, which has been reported to be closely associated with vasculature development and angiogenesis [11–13]. ROS, such as superoxide (O_2_^−•^), hydroxide (OH^−•^), and hydrogen peroxide (H_2_O_2_), are highly reactive molecules produced by the reduction of molecular oxygen. Excessive ROS cause oxidative stress (OS) and affect tissue function as a result of lipid peroxidation, protein and amino acid modification and DNA oxidation [14, 15]. Generally, OS is presumed to be implicated in diseases of placental origin. For example, redox disorders are associated with early-onset PE [16]. Although antioxidant administration to a rodent PE model [17] and pregnant women diagnosed with PE [17, 18] or FGR [19] alleviated clinical manifestations, the administration of antioxidant therapy before the onset of clinical signs did not prevent PE development, as reported by several clinical randomized controlled trials [20–23].

Previous work in our lab reported that mild OS induced by H_2_O_2_ stimulates trophoblast invasion; therefore, antioxidant treatment during placentation compromises trophoblast cell function and leads to poor placentation and subsequent adverse pregnancy outcomes, such as miscarriage, FGR, and PE [24]. Intriguingly, these diseases were also correlated with poor angiogenesis in the placenta [5, 25, 26]. However, the involvement and mechanism underlying the effect of ROS on regulating placental angiogenesis have yet to be clearly defined. Here, we investigated the role of ROS in placental development from the perspective of redox signaling.

## 2. Materials and Methods

### 2.1. Human Placentas

Patients with other major pregnancy complications, such as gestational diabetes mellitus, fetal growth restriction, spontaneous abortion, renal disease, or preeclampsia, were excluded. First trimester villi were collected from subjects who legally and voluntarily terminated their pregnancy between 6 and 10 weeks of gestational age for reasons not related to medical issues. Term placentas were collected from patients who underwent selective cesarean section. The clinical characteristics of the patients are listed in Table 1. This study was approved by the Ethics Committee of the First Affiliated Hospital of Chongqing Medical University and was conducted in accordance with the principles outlined in the Declaration of Helsinki. Written informed consent was obtained from all participants.

### 2.2. Animals

Institute of Cancer Research (ICR) mice were purchased from Huafukang Bioscience Co., Inc. (Beijing, China.). Animals were housed individually in a specific, pathogen-free environment at 25 °C on a 12 : 12 h light–dark cycle, with free access to standard mouse chow and water. Body weight and condition were monitored daily. Female ICR mice (8–10 weeks of age) were mated with males (8–12 weeks of age) to induce pregnancy (gestational day (GD) 0.5, vaginal plug). All animal experiments described in this study were conducted in accordance with the National Institutes of Health guidelines for the use and care of animals and were approved by the Institutional Animal Care and Use Committee of Chongqing Medical University. Mice were sacrificed in a CO_2_ chamber unless specifically indicated otherwise. The morphology and quantity of offspring were recorded, and related samples were collected for subsequent use.

### 2.3. Gavage

Mitoquinone mesylate (MitoQ, Antipodean Pharmaceuticals, New Zealand) dissolved in sterile water was administered to mice at a concentration of 100 *μ*mol/kg via oral gavage daily from GD 7.5 to GD 11.5. The same volume of water was administered as a vehicle control, and these mice were designated the “vehicle” group.

### 2.4. Establishment of the Mouse Model of mFlt-1-Induced PE

An adenovirus carrying mFlt-1 and empty vector (NC group) as the adenovirus control were prepared and titered by Hanbio Biotechnology Co., Ltd. (Shanghai, China). Pregnant ICR mice were divided randomly into 3 groups on GD 8.5 and injected with an adenovirus carrying mFlt-1 (10^9^ plaque-forming units in 100 *μ*L; mFlt-1 group), adenovirus carrying the empty vector (10^9^ plaque-forming units in 100 *μ*L), or the same volume of saline solution (100 *μ*L; NS group) via the tail vein.

### 2.5. Measurement of Blood Pressure

Blood pressure (BP) was measured using tail-cuff plethysmography (Visitech Systems, USA) in the morning. The mice were conscious and maintained in restrainers, with 10-20 actual measurements obtained after normalization.

### 2.6. Hematoxylin-Eosin (H&E) Staining

H&E staining was performed as previously established [27]. Briefly, fetoplacental units and kidneys were fixed with 4% paraformaldehyde, embedded in paraffin, and cut into 3-*μ*m-thick sections. The sections were dewaxed in xylene for 30 min and dehydrated in alcohol solutions with gradually decreasing concentrations. After nuclear staining with hematoxylin for 5 min and eosin for another 5 min, sections were differentiated with 1% hydrochloric acid alcohol for 3-5 s and flushed under flowing water for several minutes. Sealed sections were observed under an EVOS FL Auto microscope (Life Technologies, WA, USA), and the number of vessels was calculated using ImageJ 5.0 software (Wayne Rasband, National Institutes of Health, USA).

### 2.7. Immunohistochemical (IHC) Staining

Sections were treated with EDTA buffer (pH 9.0) for antigen retrieval and incubated with 3% H_2_O_2_ to neutralize endogenous peroxidases as previously described [28]. Primary antibodies against 4-hydroxynonenal (4HNE, 1 : 200, #ab6545, Abcam, UK), malondialdehyde (MDA, 1 : 100, #CAU27565, Biomatik, Canada), and CD31 (1 : 2000, #182981, Abcam, UK) were used. Signals were developed with diaminobenzidine (DAB staining, ZSGB-BIO, China) and observed under an EVOS FL Auto microscope.

### 2.8. Periodic Acid–Schiff (PAS) Staining

Kidneys were fixed with paraformaldehyde, embedded in paraffin, cut into sections at a 3-*μ*m thickness, and subjected to PAS staining. Glomerular open capillary areas were measured as a percentage of the glomerular tuft area. A total of 10–15 randomly selected glomeruli from each mouse were analyzed with ImageJ 5.0 software.

### 2.9. Western Blotting

Western blotting was performed as previously described. Polyvinylidene difluoride membranes (Merck Millipore, GER) were blocked for 1 h with 5% nonfat dried milk in Tris-buffered saline containing 0.05% Tween-20 (TBST) and then probed with rabbit polyclonal antibodies against 4HNE, VEGFR1, VEGFR2, AKT, pAKT, ERK, or pERK or mouse monoclonal antibodies specific for *β*-actin (1 : 1000, #3700, Cell Signaling Technology, USA) or GAPDH (1 : 1000, #ab8245, Abcam, UK) overnight at 4 °C. The membranes were then incubated with horseradish peroxidase-conjugated goat anti-mouse IgG (1 : 5000, #SA00001-1, Proteintech, USA) or goat anti-rabbit IgG (1 : 5000, #SA00001-2, Proteintech, USA) for 1 h at room temperature. Band densitometry was performed using the Quantity One System image analyzer (Bio–Rad, USA).

### 2.10. SOD and GPx Activity Assays

The enzymatic activity of superoxide dismutase (SOD) and glutathione peroxidase (GPx) was assessed using a CuZn/Mn-SOD assay kit and total glutathione peroxidase assay kit (Beyotime Biotechnology, Shanghai, China), respectively, according to the manufacturer's protocols. Briefly, villi and placental tissues were homogenized with an electric homogenizer (T8 Ultra-Turrax, IKA, GER) in a 5× volume of RIPA buffer (Beyotime Biotechnology, China) containing phenylmethanesulfonyl fluoride (PMSF, 1 : 100, Beyotime Biotechnology, China) on ice. The homogenates were then centrifuged at 13,000 × *g* for 15 min at 4 °C, and the supernatants were collected to measure the protein concentration with a bicinchoninic acid (BCA) protein quantification kit (Beyotime Biotechnology, China). After an incubation for 30 min in water-soluble tetrazolium salt (WST) working buffer at 37 °C, the absorbance of the samples was measured with a microplate reader (Thermo Fisher, USA) at 450 nm. For the analysis of GPx activity, the temperature was adjusted to 25 °C as previously described [29], and the absorbance value was measured with a microplate reader at 340 nm every 4 min for 20 min. Total GPx enzyme activity was calculated as follows:

(1) $$\begin{array}{c} \text{Total GPx enzyme activity}=\frac{\Delta \text{A}340/\min}{\varepsilon \mu \text{M}\times \text{L}\left(\text{cm}\right)}\times \frac{\text{dil}\times \left(\text{V}\left(\text{ml}\right)/\text{Vsample}\left(\text{ml}\right)\right)}{\text{protein concentration}}=\frac{\Delta \text{A}340/\min}{0.00622/\left(\mu \text{M}∙\text{cm}\right)\times 0.276\left(\text{cm}\right)}\times \frac{\text{dil}\times \left(0.1\left(\text{ml}\right)/0.02\left(\text{ml}\right)\right)}{\text{protein concentration}} \end{array}$$

ΔA340 = ΔA340 (sample) − ΔA340 (blank)

*dil* Stand for the dilution factor of samples.

### 2.11. Soluble fms-Like Tyrosine Kinase-1 (sFlt-1) Measurement

Serum was measured using an ELISA Kit for Vascular Endothelial Growth Factor Receptor 1 (VEGFR1) (# SEB818Mu, Cloud-Clone Corp. China) according to the manufacturer's instructions. Optical absorbance was read at 450 nm, and sFlt-1 concentrations were calculated from standard curves.

### 2.12. Cell Culture and Treatments

Human umbilical vein endothelial cells (HUVECs), which were purchased from Shanghai Institute of Cell Biology, Chinese Academy of Sciences, were cultured in RPMI 1640 supplemented with L-glutamine (#11875093, Gibco, USA) and 10% FBS (#ST30-2602, PAN, GER) and incubated at 37 °C with 5% CO_2_ in a humidified chamber. H_2_O_2_ (#323381, Sigma, USA) was diluted in double-distilled water, while MitoQ (#HY-100116A, MCE, USA), MK2206 (AKT inhibitor, #S1078, Selleck, China), and FR180204 (ERK1/2 inhibitor, #S7524, Selleck, China) were diluted in dimethyl sulfoxide (DMSO, Sigma, USA).

### 2.13. ROS Measurement

Intracellular ROS levels were assessed using the DCFH-DA probe (#S0033S, Beyotime Biotechnology, China) as previously described [30]. Briefly, cells were seeded onto 96-well microplates (5000 cells/well) and incubated with each of the compounds for 24 h after adhesion. The cells were then incubated with the DCFH-DA probe (1 : 1000) in RPMI 1640 medium for 20 min and washed twice with PBS. The fluorescence intensity was measured with a fluorescence microplate reader (Thermo Fisher, USA).

### 2.14. CCK8 Assay

HUVECs were seeded onto 96-well plates at a density of 5000 cells/well. All treatments were applied after cell adhesion. The medium was discarded after 24 h of treatment. Next, RPMI 1640 medium (Gibco, USA) supplemented with 10% fetal bovine serum (FBS, PAN-biotech, Germany), 100 U/mL penicillin, and 100 *μ*g/mL streptomycin (Beyotime, Shanghai, China), and containing 10% CCK-8 assay reagent (Dojindo, Japan) was added to the plates at 100 *μ*l/well and incubated for 4 h of incubation; then, the absorbance was measured at 450 nm with a microplate reader (Thermo Fisher, USA).

### 2.15. Tube Formation Assay

Matrigel (BD Biosciences, Bedford MA) was used to precoat the wells of 48-well plates. HUVECs were resuspended in medium with dissolved compounds and then seeded onto Matrigel-precoated wells (55,000 cells/well). Images were captured after 6 h of incubation. Mesh counts and the mean mesh size were analyzed with the angiogenesis analysis program in ImageJ 5.0 software (Wayne Rasband, National Institutes of Health, USA).

### 2.16. Statistics

Statistical analyses were performed using GraphPad Prism 7 software (GraphPad Software, San Diego, CA). Data in the bar graphs are presented as the means ± standard errors of the means (SEM). Comparisons among groups were analyzed with one-way or two-way ANOVA or an unpaired *t* test, as appropriate. The results with *P* < 0.05 were considered statistically significant.

## 3. Results

### 3.1. Placental ROS Levels Peak in Early Gestation

**Figure 1:**
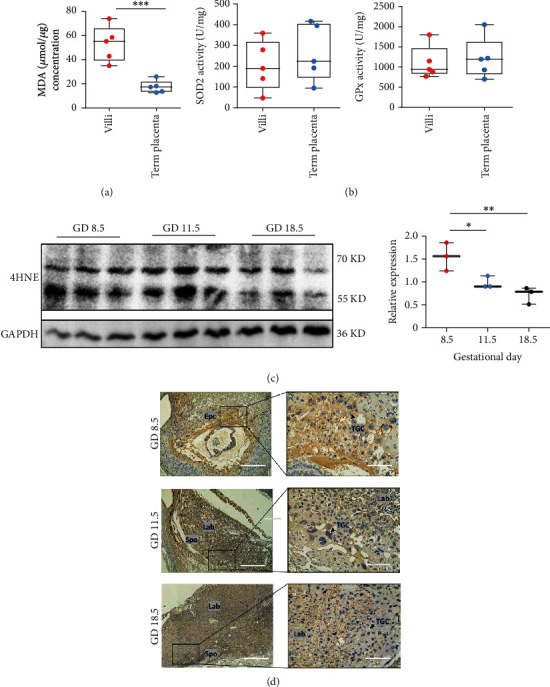
Placental ROS levels peak in early gestation and gradually decrease as pregnancy proceeds. (a) MDA levels in human first trimester villi and term placentas, *n* = 5. Data were analyzed using unpaired Student's *t* test (*t* test). (b) SOD2 and GPx enzyme activity assays in human first trimester villi and term placentas, *n* = 5. Data were analyzed using a *t* test. (c) Western blot showing 4HNE levels in the sac and placenta of normal pregnant mice collected on GD 8.5, GD 11.5, and GD 18.5, *n* = 3 mice per group. Data were analyzed using one-way ANOVA, followed by Sidak's multiple comparison tests. (d) IHC staining for 4HNE in frozen sections of mouse fetoplacental units collected on GD 8.5, GD 11.5, and GD 18.5. Abbreviations: TGC: trophoblast giant cell; EPC: ectoplacental cone. Scale bars: 100 *μ*m. Data are presented as the means ± SEM. ∗*P* < 0.05, ∗∗*P* < 0.01, and ∗∗∗*P* < 0.001.

MDA levels in human placenta collected from villi and term placentas were measured to determine placental ROS production across gestation. MDA levels were higher in villi and reduced in late gestational stages (Figure 1(a)). Meanwhile, the activities of GPx and SOD did not differ between early villi and term placenta (Figure 1(b)). These facts indicate a burst of ROS production in placentas during early gestation.

Consistent with the results obtained from the human placenta, 4HNE levels were significantly reduced from GD 8.5 to GD 18.5 in mouse placentas (Figure 1(c)). Interestingly, 4HNE staining was restricted to the ectoplacental cone (EPC) on GD 8.5, with smeared staining observed in the labyrinth near trophoblast giant cells (TGCs), and the staining gradually decreased from GD 11.5 to GD 18.5 (Figure 1(d)). Taken together, high placental ROS levels coincident with placentation implies that they may be required for placental development.

### 3.2. Eliminating ROS During Placentation Disrupt the Placental Vasculature in Mice

**Figure 2:**
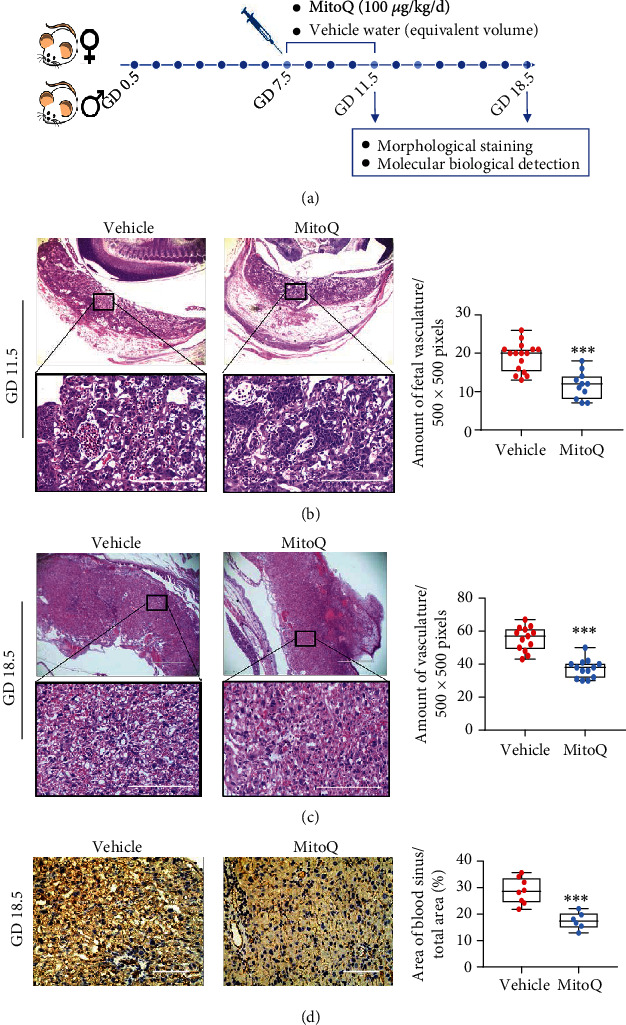
Eliminating ROS during placentation results in placental malformation in mice. (a) Diagram of the experimental design. (b, c) H&E staining of the mouse placental labyrinth on GD 11.5 (b) and GD 18.5 (c). Scale bars: 1000 *μ*m (upper panel) and 200 *μ*m (lower panel). The number of fetal vessels was counted per 500 × 500 pixels. *n* = 3 mice per group; (d) IHC staining for CD31 in the mouse placental labyrinth on GD 18.5 after treatment with/without MitoQ. The density of blood sinuses in the labyrinth was quantified, with scale bars representing 100 *μ*m. Data are presented as the means ± SEM. Data were analyzed using unpaired Student's *t* test (t test). ∗*P* < 0.05, ∗∗*P* < 0.01, and ∗∗∗*P* < 0.001.

Pregnant ICR mice were orally administered the potent antioxidant MitoQ (100 *μ*g/kg/d) from GD 7.5 to GD 11.5 to investigate the involvement of ROS in placentation. Fetoplacental units were collected on GD 11.5 and GD 18.5 (Figure 2(a)). H&E staining revealed that the intervillous space in the placental labyrinth developed on GD 11.5 (Figure 2(b)), and the vascular density increased significantly by GD 18.5 (Figure 2(c)). On GD 11.5, fetal vessels were distinguished from maternal vessels based on the nucleated erythrocyte in the murine labyrinth; however, the number of fetal blood vessels was reduced when MitoQ was administered from GD 7.5 to GD 11.5 (Figure 2(b)). Similarly, the number of vessels was also significantly reduced in the MitoQ group compared with the vehicle group on GD 18.5 (Figure 2(c)). Consistently, the administration of MitoQ from GD 7.5 to GD 11.5 significantly reduced the density of the placental blood sinus (Figure 2(d)). These data suggest that the suppression of ROS production during placentation might compromise neovascularization in the placenta.

### 3.3. Suppression of ROS Production During Placentation Compromises Angiogenic Signaling in the Mouse Placenta

**Figure 3:**
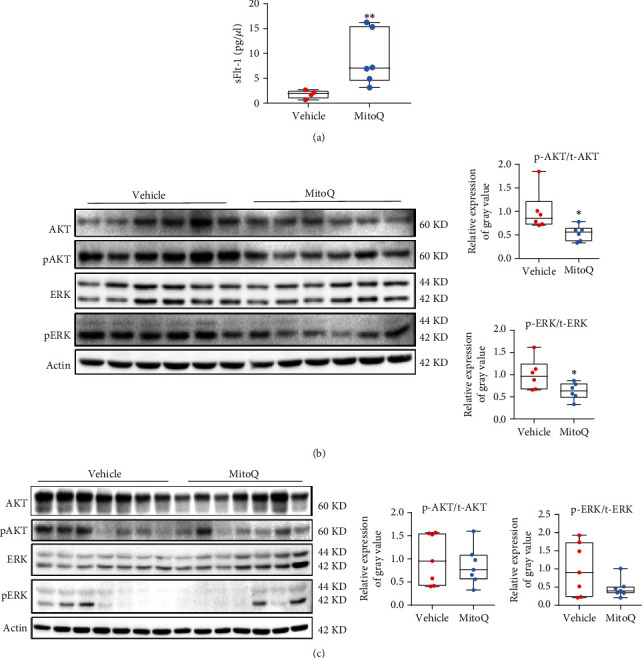
Scavenging ROS during placentation disturbs angiogenic signaling in the mouse placenta. (a) Serum sFlt-1 levels in the vehicle and MitoQ groups on GD 18.5, as measured using ELISA. *n* = 6 mice per group. (b, c) Western blots showing the levels of proteins involved in AKT and ERK signaling in placentas from mice administered water or MitoQ on GD 11.5 (b, *n* = 6 mice per group) and GD 18.5 (c, *n* = 7 mice per group). Data are presented as the means ± SEM. Student's *t* test was used to analyze the data. ∗*P* < 0.05 and ∗∗*P* < 0.001.

We first measured serum sFlt-1 levels to further investigate the underlying molecular mechanism of ROS-induced placental angiogenesis and found that MitoQ treatment from GD 7.5 to GD 11.5 resulted in a significant increase in sFlt-1 levels in late gestation (Figure 3(a)). Considering the well-documented anti-angiogenic effect of sFlt-1 [31], we then assessed the activity of AKT and ERK in mouse placentas collected on GD 11.5 and GD 18.5, respectively (Figures 3(b) and 3(c)). Dephosphorylation of AKT was observed only in the MitoQ group on GD 11.5, while ERK phosphorylation was significantly downregulated in the MitoQ group on GD 11.5, showing a decreasing trend on GD 18.5 (Figures 3(b) and 3(c)). Taken together, the disturbance of angiogenic signaling and subsequent compromised neovascularization in the mouse placenta induced by MitoQ may be attributed to sFlt-1.

### 3.4. Placental Angiogenesis Is Impaired in a Mouse PE Model

**Figure 4:**
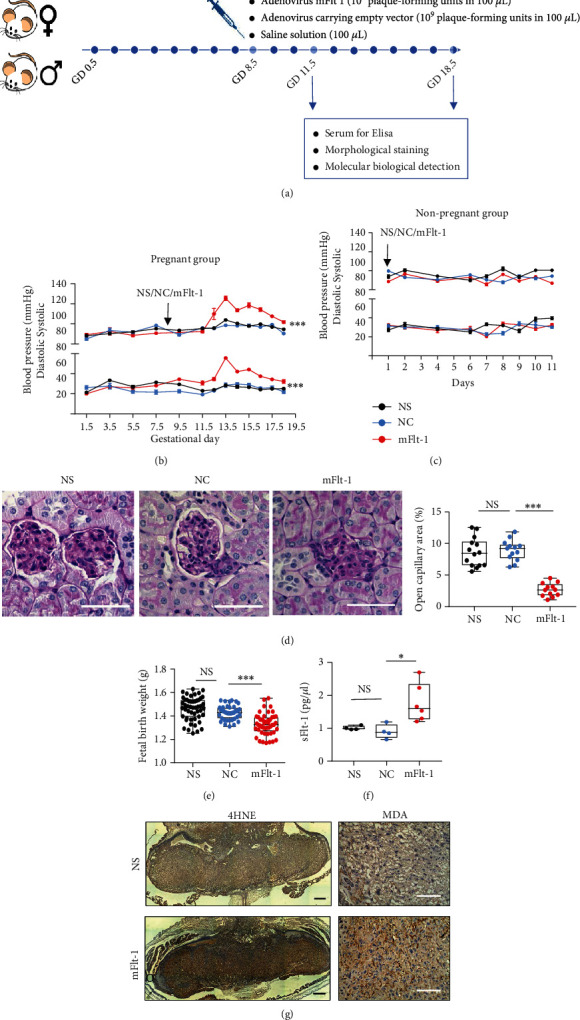
Overexpression of Flt-1 induces a PE-like phenotype in mice. (a) Mating and treatment plan for the establishment of the PE mouse model. (b, c) Systolic and diastolic BP of pregnant mice (b) and nonpregnant mice (c) measured using a tail vein cuff. (d) PAS staining of the kidney and quantification of the glomerulus open capillary area in different groups (*n* = 3 mice per group); scale bars: 50 *μ*m. (e) Fetal birth weight. NS group: *n* = 50 pups from 4 dams (50/4), NC group: *n* = 43/4, mFlt-1: *n* = 47/5. F. Serum sFlt-1 levels in the NS, NC, and mFlt-1 groups on GD 11.5, as measured using ELISA. *n* = 6 mice per group. (g) IHC staining for 4HNE (upper panel) and MDA (lower panel) in the mouse placenta on GD 18.5 in the NS and mFlt-1 groups. Scale bars: upper panel -1 mm and lower panel -100 *μ*m. Data are presented as the means ± SEM. Data were analyzed using two-way (b, c) and one-way (d, e, f) ANOVA, followed by Sidak's multiple comparison tests. ∗*P* < 0.05, ∗∗*P* < 0.01, and ∗∗∗*P* < 0.001.

Mice were injected with an adenovirus carrying mFlt-1 (10^9^ plaque-forming units in 100 mL; mFlt-1 group), an adenovirus carrying the scrambled fragment (10^9^ plaque-forming units in 100 mL; NC group used as a control for the virus), or saline solution (100 mL; NS group) through the tail vein on GD 8.5 to determine whether the inhibition of ROS production replicated the effect of elevated sFlt-1 levels on placental angiogenesis (Figure 4(a)). Our results showed that systolic and diastolic BP increased significantly from GD 12.5 to GD 18.5 in mice that received the mFlt-1 adenovirus injection compared with the NC and NS groups (Figure 4(b)). However, the BP of nonpregnant mice was not affected (Figure 4(c)). Meanwhile, PAS staining of kidney sections from dams showed that the glomerulus open capillary area was remarkably reduced in the mFlt-1 group (Figure 4(d)). Consistently, the fetal birth weight was significantly decreased by the mFlt-1 adenovirus treatment compared with controls (Figure 4(e)). ELISA also ascertained markedly increased serum sFlt-1 levels on GD 11.5 in the mFlt-1 group (Figure 4(f)). Consistent with a previous report that elevated sFlt-1 levels result in increased oxidative stress, we observed significantly higher 4HNE and MDA levels in the placentas from mFlt-1 mice than in those from NS mice (Figure 4(g)).

**Figure 5:**
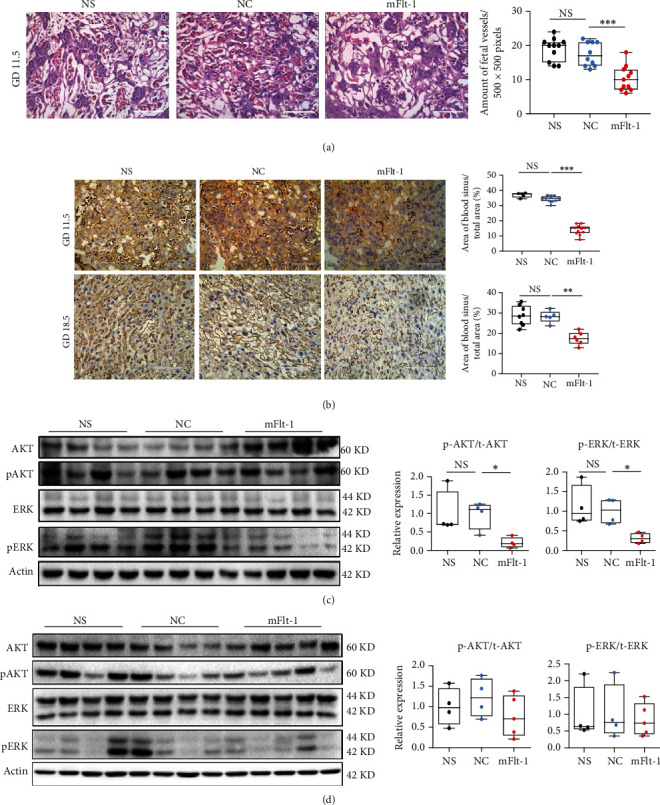
Flt-1 induced PE and impaired angiogenesis in the murine placenta. (a) H&E staining of the mouse placental labyrinth collected on GD 11.5. Scale bars: upper panel -1000 *μ*m and lower panel -200 *μ*m. The number of fetal vessels was counted per 500 × 500 pixels. *n* = 3 mice per group. (b) IHC staining for CD31 in the placental labyrinth on GD 11.5 and GD 18.5 from mice treated with mFlt-1, NC adenovirus, or NS injection. The area of blood sinuses in the labyrinth was quantified, with scale bars indicating 100 *μ*m. (c, d) Western blots showing the levels of proteins related to AKT and ERK signaling in the placentas of mice treated with mFlt-1, NC adenovirus, or NS injection on GD11.5 (c, *n* = 4 mice per group) and GD18.5 (d, *n* = 4 mice in the NS and NC groups, *n* = 5 mice in the mFlt-1 group). Data are presented as the means ± SEM. Data were analyzed using one-way ANOVA, followed by Sidak's multiple comparison tests. ∗*P* < 0.05, ∗∗*P* < 0.01, and ∗∗∗*P* < 0.001.

Along with the increase in serum sFlt-1 levels, the density of fetal blood vessels was reduced in the mFlt-1 group (Figure 5(a)), similar to our findings from the MitoQ group. In addition, IHC staining of murine placentas collected on both GD 11.5 and GD 18.5 showed that CD31 expression was significantly downregulated in the mFlt-1 group compared to the NC and NS groups (Figure 5(b)). This evidence strongly indicates that mouse placental vascularization is noticeably impaired by elevated sFlt-1 levels during placentation.

Meanwhile, related angiogenic signaling in placentas was determined using western blotting. The ratios of p-AKT/t-AKT and p-ERK/t-ERK were both significantly decreased in the mFlt-1 group on GD 11.5 but not on GD 18.5 (Figures 5(c) and 5(d)). Based on these results, either reducing ROS levels or increasing circulating sFlt-1 levels during placentation compromises angiogenesis in murine placentas, possibly by suppressing AKT and ERK signaling.

### 3.5. Certain Levels of ROS Stimulate the Proliferation and Angiogenesis of HUVECs

**Figure 6:**
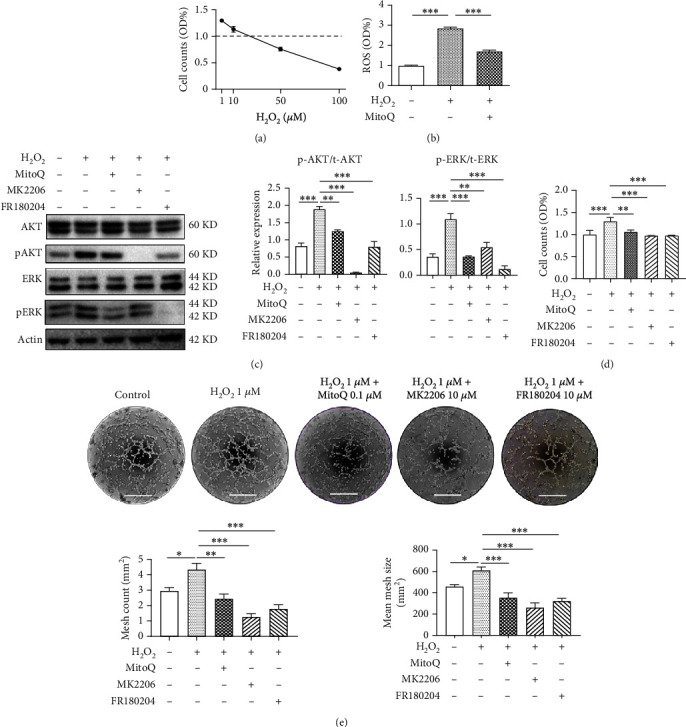
Moderate ROS levels stimulate the proliferation and angiogenesis of HUVECs. (a) CCK-8 assay of HUVECs treated with various doses of H_2_O_2_. (b) HUVECs were treated with 1 *μ*M H_2_O_2_ alone or with 0.1 *μ*M MitoQ for 24 h and then subjected to staining with the DCFH-DA probe. (c) HUVECs were treated with 1 *μ*M H_2_O_2_ alone or in combination with 0.1 *μ*M MitoQ, 10 *μ*M MK2206 (AKT inhibitor), or 10 *μ*M FR180204 (ERK1/2 inhibitor) for 24 h, and then AKT and ERK signaling were detected using Western blotting. (d) Cell counts determined using the CCK-8 assay. (e) Tube formation by HUVECs after treatment with 1 *μ*M H_2_O_2_ for 6 h in the presence and absence of antioxidants, AKT inhibitor (MK2206), or ERK1/2 inhibitor (FR180204). The statistical analysis of the mesh count and mean mesh size are shown below. Scale bars: 400 *μ*m. *n* = 4 samples per group. Data in bar graphs are presented as the means ± SEM. The data were analyzed using one-way repeated-measures ANOVA, followed by Sidak's multiple comparison tests. ∗*P* < 0.05, ∗∗*P* < 0.01, and ∗∗∗*P* < 0.001.

Several doses of H_2_O_2_ were applied to HUVECs for 24 h to elucidate the underlying mechanism by which ROS regulate placental angiogenesis. A lower dose (1 *μ*M and 10 *μ*M) of H_2_O_2_ increased cell numbers, while excessive H_2_O_2_ (50 *μ*M and 100 *μ*M) resulted in cell loss in a dose-dependent manner (Figure 6(a)). Intracellular ROS levels were increased approximately 2.5-fold following the administration of 1 *μ*M H_2_O_2_ compared to controls; this increase in ROS levels was largely alleviated by supplementation with 0.1 *μ*M MitoQ (Figure 6(b)).

Since AKT and ERK signaling in HUVECs was suppressed by MitoQ treatment, we then determined whether the AKT and ERK signaling pathways were involved in ROS-induced HUVEC proliferation. An AKT inhibitor, MK2206, and an ERK1/2 inhibitor, FR180204, were applied to HUVECs in the presence of 1 *μ*M H_2_O_2_ with or without MitoQ. H_2_O_2_ treatment alone substantially increased AKT and ERK phosphorylation, whereas MitoQ, MK2206, or FR180204 all significantly abolished the activation of AKT and ERK (Figure 6(c)). CCK-8 assays also revealed that the improvement in HUVEC proliferation induced by 1 *μ*M H_2_O_2_ was largely blocked by MitoQ, MK2206, or FR180204 (Figure 6(d)). Next, we evaluated the effect of ROS on the angiogenic capacity of HUVECs. The data showed that 1 *μ*M H_2_O_2_ noticeably increased the mesh count and mean mesh size, which were completely blunted by the addition of MitoQ, MK2206, or FR180204 (Figure 6(e)).

## 4. Discussion

Normal development of the placental vascular network requires angiogenesis, vasculogenesis, and spiral artery remodeling [32]. Abnormal angiogenesis in the placenta leads to placental dysfunction, thereby resulting in the blockage of nutrient and waste exchange between the mother and fetus [33]. Emerging evidence suggests that angiogenesis is stimulated by ROS derived from ECs and other cell types [34]. In endothelial cells, ROS play a key role in the angiogenic response induced by growth factors such as VEGF [35]. Excess ROS contribute to pathological angiogenesis involved in cancer, atherosclerosis [36], and pathological retinopathy [34]. Yang et al. reported that the elimination of ROS during placentation impairs placental development and induces PE-like symptoms in mice, which is attributed to improper trophoblast invasion [24]. However, the involvement of ECs in the regulatory effects of ROS on placental development remains unknown.

We first determined the level of oxidative stress in the placenta across gestation and found that ROS levels gradually decreased as gestation continued, consistent with reports of proteomics analyses showing that proteins involved in the thiol/disulfide oxidoreductase system were upregulated in human first-trimester placentas [37]. Raijmakers et al. illustrated an approximately three times higher level of NAD(P)H oxidase (Nox), which is a major source of ROS-mediated superoxide generation, in early pregnancy placental tissue than in placental tissue collected at full term [38]. Moreover, Hernandez and colleagues showed a significantly higher protein level of the p47phox subunit, which has an organizing role in the regulation of Nox activity in the chorionic villi, in the early stage in the first trimester [39]. This evidence indicates that ROS may be critical for placental development in early gestation.

Placental angiogenesis is strongly stimulated in early pregnancy, while angiogenesis must be progressively limited with placental decay in late pregnancy [40]. We reported that the administration of the mitochondrial targeting antioxidant MitoQ in mice during placentation resulted in a hypovascularized labyrinth. Similarly, Nezu et al. reported that enhancing cellular antioxidant responses in mice by activating Nrf2 represses angiogenesis in the placenta [41]. In addition, AKT and ERK signaling was significantly reduced by MitoQ on GD 11.5 in the present study. Collectively, these findings indicate that ROS stimulate placental angiogenesis by activating AKT and ERK signaling during placentation.

In addition, ROS-induced oxidative stress initiates the secretion of angiogenic modulators, such as VEGF and HIF-1*α* [42–44], which have been shown to promote the growth of fetoplacental vessels. Interestingly, bevacizumab, an anti-VEGF antibody that decreases VEGF signaling in a manner similar to sFlt-1, causes hypertension and proteinuria in nonpregnant individuals [45–47]. In the present study, Flt-1 overexpression in mice during placentation led to PE-like symptoms, including compromised angiogenesis in the labyrinth, and induced hypertension, kidney damage, and FGR. These manifestations are very similar to those caused by MitoQ treatment during early pregnancy, implying that ROS are involved in PE pathogenesis through the regulation of placental angiogenesis.

HUVECs were treated with H_2_O_2_, a major source of ROS implicated in several diseases and thus commonly used as an ROS inducer, to study the underlying mechanism of ROS-stimulated angiogenesis [48–50]. Our results showed that high ROS levels significantly increased the proliferation and angiogenesis of HUVECs, while the addition of the antioxidant MitoQ abolished these effects. Indeed, although ROS directly damage proteins, lipids, and nucleic acids, overproduction of H_2_O_2_ causes oxidative stress and is a hallmark of vascular diseases [51, 52]. Based on accumulating evidence, moderate H_2_O_2_ levels might be beneficial and/or protective under many physiological conditions [53]. For instance, H_2_O_2_ has been well recognized as a critical mediator not only of vascular smooth muscle cell (VSMC) function under physiological conditions [54] but also for regulating coronary blood flow (CBF) [55]. These facts imply that lower levels of ROS function as signaling molecules to adapt to stress. Even lower levels of ROS are required for normal cell homeostasis.

HUVECs, which are derived from the endothelium of human umbilical cord veins, play a major role as a model system in studies of the regulation of EC function [56]. Consistent with previous reports [52, 57], the present study proposes that moderate H_2_O_2_ levels increase the proliferation and angiogenesis of HUVECs, while excess H_2_O_2_ is toxic. ROS are closely correlated with angiogenesis in tumors [34]. During the initial period of tumorigenesis, new blood vessels develop from the preexisting vasculature through a process known as angiogenesis that supports tumor proliferation and survival [58–60]. ROS-dependent angiogenesis is initiated through tumor proliferation, which in turn increases the metabolic rate, leading to the generation of high ROS levels. This process is extremely similar to the development of the placenta, which also experiences increasing ROS production from hypoxia to reoxygenation [61].

## 5. Conclusions

Taken together, moderate ROS levels may meet a physiological demand to support EC function in placentation. Eliminating ROS may disturb placental angiogenesis and increase the risk of PE development. Therefore, based on our findings, physicians should carefully consider prescribing antioxidants to women with typical pregnancies in the first trimester.

## Figures and Tables

**Table 1:** The clinical characteristics of the study population.

Parameters	First trimester (*n* = 10)	Third trimester (*n* = 10)	*P* value
Maternal age	32 ± 3.2	32.4 ± 3.6	0.8026
Gestational age at delivery (weeks)	7.8 ± 1.2	37.8 ± 2.2	≤0.0001
Body mass index	21.1 ± 2.9	22.5 ± 3.6	0.3889
Systolic pressure (mmHg)	115 ± 10.1	113.8 ± 6.4	0.7551
Diastolic pressure (mmHg)	69.1 ± 6.5	71.8 ± 6.1	0.3513

## Data Availability

The datasets used or analyzed during the current study are available from the corresponding author upon reasonable request.
